# Optogenetics for Understanding and Treating Brain Injury: Advances in the Field and Future Prospects

**DOI:** 10.3390/ijms23031800

**Published:** 2022-02-04

**Authors:** Yuwen Sun, Manrui Li, Shuqiang Cao, Yang Xu, Peiyan Wu, Shuting Xu, Qian Pan, Yadong Guo, Yi Ye, Zheng Wang, Hao Dai, Xiaoqi Xie, Xiameng Chen, Weibo Liang

**Affiliations:** 1 West China School of Basic Medical Sciences and Forensic Medicine, Sichuan University, Chengdu 610041, China; 2017151611001@stu.scu.edu.cn (Y.S.); 19944501946a@gmail.com (Y.X.); mdsxwu2002@163.com (P.W.); 2021151610079@stu.scu.edu.cn (S.X.); panqian1078@scu.edu.cn (Q.P.); 2 Department of Forensic Genetics, West China School of Basic Medical Sciences and Forensic Medicine, Sichuan University, Chengdu 610041, China; marylee078@gmail.com (M.L.); caoshuqiang916@163.com (S.C.); 3 Department of Forensic Science, School of Basic Medical Sciences, Central South University, Changsha 410013, China; gyd82@126.com; 4 Department of Forensic Toxicological Analysis, West China School of Basic Medical Sciences & Forensic Medicine, Sichuan University, Chengdu 610041, China; yeyiconan@163.com; 5 Institute of Forensic Medicine, West China School of Basic Science and Forensic Medicine, Sichuan University, Chengdu 610041, China; wangzhengtim@scu.edu.cn; 6 Department of Forensic Pathology and Forensic Clinical Medicine, West China School of Basic Medical Sciences and Forensic Medicine, Sichuan University, Chengdu 610041, China; daihao22@gmail.com; 7 Department of West China Clinical Medicine, Sichuan University, Chengdu 610041, China; xiaoqixie1898060@163.com

**Keywords:** opsins, traumatic brain injury, neural circuitry, neuronal apoptosis, neuroregeneration

## Abstract

Optogenetics is emerging as an ideal method for controlling cellular activity. It overcomes some notable shortcomings of conventional methods in the elucidation of neural circuits, promotion of neuroregeneration, prevention of cell death and treatment of neurological disorders, although it is not without its own limitations. In this review, we narratively review the latest research on the improvement and existing challenges of optogenetics, with a particular focus on the field of brain injury, aiming at advancing optogenetics in the study of brain injury and collating the issues that remain. Finally, we review the most current examples of research, applying photostimulation in clinical treatment, and we explore the future prospects of these technologies.

## 1. Introduction

Optogenetics is a novel technique that incorporates the knowledge of bioengineering, genetics, optics, virology, and neuroscience [1]. Optogenetics combines genetic manipulation with optical stimulation, making target cells obtain or lose specific functions in vivo [2]. The applications and achievements of optogenetics benefit from the development of its core technologies, such as the discovery of additional microbial opsin variants, development of opsin targeting strategies, and advancement of optical targeting devices. Further, the integration of auxiliary technologies, such as data visualization using electrophysiology and neuron activity imaging, gives optogenetics the marked advantage of reflecting cellular function on different temporal and spatial scales [1]. The combinations of these technologies are powerful tools for analyzing the abnormalities of neural circuits in brain diseases, such as Parkinson’s disease [3], epilepsy [4], pain [5], etc., thereby promoting their understanding and treatment. Further development of these technologies will likely contribute to revolutions in neuroscience research.

To date, numerous studies have focused on elucidating complex neural processes and treating neuropsychiatric diseases [6,7,8], but few studies have systematically reviewed the latest studies to examine the potential use of optogenetics to map and treat brain injury. Brain injury places a significant health burden on individuals and communities [9,10,11,12,13]. After a primary injury is caused by bleeding, vascular occlusion, or mechanical forces, secondary alterations such as consequent axonal shearing, blood–brain barrier (BBB) disruption, etc., may lead to aggravated neuronal dysfunction and cell death [14,15], amplifying the negative outcomes of acute brain injury (ABI). Owing to the complicated pathophysiology and various clinical characteristics of brain injury, post-acute treatment options are limited. Numerous potential treatment methods have been explored in multiple animal models, including transcranial magnetic stimulation, electrical cortical stimulation, deep brain stimulation, pharmacological approaches [16,17], etc. However, few studies have provided evidence of therapeutic potential in human trials. Limitations of these treatments include that stimulation therapies may provide temporal control of neurons but often lack spatial specificity, pharmacological therapies exhibit spatial specificity but lack precise temporal control, etc.

Lately, researchers are incorporating single-cell control and millisecond precision timing into the development of treatment options using optogenetics. Optogenetics is superior to conventional methods in some respects because of more precise spatiotemporal control of cellular processes and the option of performing parallel investigations at multiple sites. Although optogenetics provides solutions to notable challenges associated with conventional neuroscience research, these methodologies still have some shortcomings that need to be innovatively addressed to enhance their future application potential [18]. This review aims to provide an overview of the latest research in the field of brain injury mapping and therapeutics using optogenetics and to summarize future development strategies that will enhance its prospects as a tool for understanding pathological conditions associated with brain injury.

## 2. Fundamentals of Optogenetics

The application and development of optogenetics allows neuroscientists to control neuronal activities with light and permits multi-level neuroscience research. To this end, the expression of light-sensitive proteins and light-mediated modulation of cellular function is critical.

### 2.1. Overviews of Opsins

Optogenetics utilizes the light-sensitive proteins (e.g., opsins) to regulate the activities of target cell populations using light stimulation. Table 1 summarizes several common optogenetic tools with distinctive characteristics. Microbial (type I) and animal (type II) opsins were long considered to be the only existing types of rhodopsins; however, a recently discovered protein family with an inverted membrane topology—the heliorhodopsin—is expected to be incorporated as a new optogenetic tool in future research [19,20].

Lately, there have been increasing concerns that currently available optogenetic tools may have low efficiency issues and off-target effects. The weak current and rapid inactivation properties of natural channelrhodopsins (ChRs) limited their clinical application. To circumvent this issue, attempts have been made to improve channel dynamics and operational light selectivity of opsins to compensate for above limitations [26,31]. One method of improving light sensitivity is opsin modification using genetic engineering, examples of which include iC1C2 (chimeras of ChR1 and ChR2) [32] and SOUL (an engineered step function opsin containing a particular combination of mutations) [33]. Another method is to search for opsins from other species or optimize existing ChR variants. Examples include the viral rhodopsins OLPVR1, VirChR1 [34] and *Chloromonas oogama* ChR [35].

Bidirectional control of neuronal activity was needed in some studies but has been challenging until last year [36]. In 2021, Vierock et al. [37] reported that a two-channel fusion protein—BiPOLES (Bidirectional Pair of Opsins for Light-induced Excitation and Silencing)—induces neuronal excitation with red light and inhibition with blue light. Until now, the BiPOLES has been utilized and verified in several animal models [37].

Except for opsins, non-opsin-based photosensory proteins has been emerging to control protein activity and increasingly meet certain experimental requirements [38,39,40]. In recent studies, fluorescein, a strongly bioluminescent protein, allows reliable activation of neural circuits at different temporal-spatial resolutions and regulation of neurons with satisfactory experimental outcomes [41,42]. Therefore, the research for new unique photoproteins alternatives as well as the assessment and optimization of existing opsin properties is valuable. In so doing, new opportunities for designing efficient internal light-inducing systems are introduced, and their clinical applications may be facilitated.

### 2.2. Light Delivery Systems

Light stimulation, targeting specific opsins, acts as an essential component of optogenetics. A laser or light-emitting diode (LED) can be coupled with conventional one-photon (1P) or two-photon (2P) imaging to observe neural activities and subsequent behavioral changes. Regardless of the method used, light scattering limits the depth of light penetration and affects 1P and 2P imaging. Furthermore, external light sources (laser or LED) can cause unexpected light-induced behaviors, such as fear responses and disruptive movement, which may affect the experimental outcomes in behavioral research. To avoid these behaviors and achieve deep-brain photostimulation, the use of invasive optical fibers may be necessary. However, such approaches pose the risk of greater or unintended damage to the brain tissue.

Classical optical fibers are planar fibers, which limit light penetration in a small region [43]. Tapered fibers allow for input angle of cylindrical light to be adjusted so that the same fiber can illuminate two different brain regions [44]. To improve the spatial accuracy, microscale LED arrays and tapered optical fibers, which reduce injury and imprecise positioning of the light sources, have been widely applied this year [44,45]. In addition, the development of nanomaterials has become a research objective for optogenetics. Nanomaterials are often used to minimize photothermal effect while optimizing light induction. As efficient light absorbers, gold–based nanomaterials have been placed near the targeted tissue, thereby avoiding thermal damage to non–targeted areas [46]. Carbon nanotubes, a stretchable transparent electrode array combining nanotechnology and optogenetics, have been used to record response signals from cortical surfaces after photostimulation. These nanotubes may potentially be used for in-depth, real-time, and continuous monitoring of disrupted cerebral cortex function [47]. Upconversion nanoparticles absorb tissue-penetrating, near-infrared light and emit wavelength-specific visible light. These characteristics allowed their use for neural stimulation in several animal models [48,49], and indicated their potential to be a substitute for invasive optical fibers [50].

Although recent efforts have greatly improved existing optogenetic devices, their potential clinical applications in human patients will require further development and careful consideration. Continuous advancements in the fields of materials science, nanotechnology, chemistry, and optics are needed for the possible application of fibreless optogenetics in neuroscience and beyond.

## 3. Optogenetics Applied to Brain Injury

In the field of brain injury research, optogenetics has been preliminarily used to induce and monitor traumatic brain injury (TBI) in animal models (Table 2). In this section, we focus on the contribution of optogenetics to the field of brain injury in recent years, including TBI, stroke, and spinal cord injury (SCI). In addition, we discuss prospects for the practical application of optogenetics for these injuries.

### 3.1. Optogenetics in Brain Monitoring

A combination of clinical assessment and imaging is commonly used to judge the severity and prognosis of brain injury [58]. Additional approaches can help identify local environmental insults. These approaches include cerebral microdialysis, cerebrovascular pressure response and pressure reactivity indices, intracranial and cerebral perfusion pressure monitoring, and identification of serum markers for neuronal damage [59,60,61,62]. These approaches are also valuable for predicting prognosis, measuring the degree of injury, and guiding intervention methods.

Optogenetics is another approach with brain–monitoring potential in cases of brain injury, which facilitates the study of pathological processes post-injury and therapeutic targets at a different level. Using optogenetic methods, the dynamic tracking of secondary changes after brain injuries are possible, offering an added advantage over current brain monitoring methods. Many experimental models in which this approach has been applied emphasize the utility of optically recording neural activity [63,64].

Optogenetics involves optically recording changes in membrane potential and observing neuronal signal transmission [65]. Therefore, functional brain research can be facilitated using this technique. Drawing inspiration from previous studies, Adams and colleagues [55] first employed optogenetic photostimulation to monitor and explicate neuronal function following TBI. They stimulated cortical pyramidal neurons in a mouse model of repeated mild TBI (mTBI) and investigated neuronal function change through bilateral intracranial electrophysiological recordings. Their experimental data suggested that optical stimulation led to reduced evoked neuronal responses and impaired functioning of the surviving neurons [55]. Another novel approach–in vivo optogenetic motor mapping, was first reported by Nguyen et al. [52]. In their study, this optogenetic method was used to evaluate longitudinal changes in cortical motor maps in an mTBI model, remedying the limitation of some conventional research techniques. Real-time performance is another advantage of optogenetics. Lately, studies successfully monitored real-time neurotransmitter release and synapse formation using optogenetics [66,67], which has been a challenge previously.

Regional abnormalities in cerebral blood flow are important to understanding the pathogenesis of brain injuries as well as monitoring the clinical course. Adams et al. has demonstrated that photostimulation of ChR2 can be used to probe neurovascular unit function with more robust vascular responses and greater spatiotemporal control than physiological stimuli [55]. After inducing ChR2 expressing in mice, Mester et al. [54] measured cerebral vessel activity and local neuronal reactivity after mTBI and found that photostimulation resulted in dilatation and augmented venular reactivity. This was the first study to integrate optogenetics, 2P fluorescence microscopy, and intracortical electrophysiological recordings for the examination of neurovascular unit dysfunction after repeated mild TBI [54]. Taken together, recent studies suggest that optogenetic techniques can complement current methods of brain monitoring and are potential tools for brain injury treatment and analysis.

### 3.2. Optogenetics in Analyzing Neural Circuitry

Neural circuits are composed of highly interconnected neurons with distinct functions which coordinate simultaneously to drive the normal operation of the nervous system. Owing to the disconnection of surviving circuits after brain injury, patients may suffer sensory or motor impairments, and the detection of functional abnormalities in specific neuronal nuclei and neural circuits might aid in the prevention, diagnosis, and treatment of these disorders. The high temporospatial resolution feature of optogenetics makes it particularly suitable for neuronal circuit manipulation, which benefits the study of neuronal clusters from multiple dimensions, the changes in abnormal neural circuits and the pathogenesis of certain diseases, such as Alzheimer’s disease [68], Parkinson’s disease [69], and Huntington’s disease [70].

Cheng et. al. report that optically targeting specific cerebral cortex regions [71] (ipsilesional primary motor cortex, iM1) and nuclei [72] (lateral cerebellar nucleus, LCN) leads to a significantly increased capacity for neuroplasticity, with elevated expression of the plasticity marker, axonal growth-associated protein 43 (GAP43). A comparison between LCN stimulation and iM1 stimulation suggests that photostimulation of the LCN may be more efficient in increasing GAP43 in the ipsilesional somatosensory cortex [72]. A recent study also showed that optogenetic stimulation of the LCN produced functional benefits in a murine stroke model and downregulated the expression of neuronal nitric oxide synthase, a key regulator of the neurovascular response in stroke, suggesting that optogenetic stimulation of the LCN holds promise for facilitating functional limb movements and behavioral recovery [73].

The optogenetic monitoring and optimization of axon connections has emerged as an advanced tool in the field of neuronal circuit study [66]. After introducing viral vectors carrying the fluorescently-tagged opsin *ChR2* transgene into TBI rats, Ndode-Ekane et al. [56] observed the reformulation of axonal projection terminals in the primary somatosensory cortex. Optogenetic photostimulation led to reduced density of axonal terminals in the cortex and hyperexcitability of thalamo-cortical network activity after TBI [56]. Similarly, another study revealed that renewed cortico-spinal tract axons can be integrated into the inferior neural circuit through optogenetics, and that the regenerated axons promoted the recovery of sensorimotor function [74]. Combined with other imaging methods, this light-sensitive technology can promote the reorganization and functional recovery of impaired neural circuits. Using in vivo calcium imaging, Tennant et al. [75] found that stroke results in disruption of axonal synaptic connections in the damaged cortex and causes sustained excitability impairment of surviving thalamocortical circuits. Further, they revealed that repeated photostimulation in the transcranial optical window accelerated the reorganization of thalamocortical synaptic boutons and the subsequent recovery of cortical circuit function for the first time [75].

Optogenetic photostimulation can be used to investigate the role of pathological processes and functional recovery post injury by targeting neuronal circuits. Memory and cognitive impairments caused by brain injury may have serious effects on survivors, and functional alterations in neural circuits are of the leading factors contributing to these impairments. To explore the relationship between impaired spatial recognition memory function and corresponding brain regions, Zeng et al. [53] optogenetically inhibited the function of the retrosplenial cortex (RSC) and observed memory impairment in wild-type mice. Currently, light stimulation alone cannot restore all brain functionality after injury, whereas the combination of optogenetics and other type of treatment may promote recovery to a greater extent. For example, optogenetic stimulation of the motor cortex combined with exercise training has been shown to facilitate neuroplasticity and restore motor function [76]. Considering the evidence, optogenetics allows for the electrical activity of individual neurons to be observed and the function of essential neural circuits to be analyzed by activating or inhibiting sites via photostimulation.

### 3.3. Optogenetics in Protecting Neural Cells

Accumulated evidence from animal and human studies demonstrates that apoptosis, autophagy, acidosis, etc., contribute to the overall pathologenesis following brain injury [77,78,79], interventions targeting these factors also show marked prospects for brain injury treatment. In 2020, Bo et al. [80] transferred protons out of penumbra neurons and explored whether the proton-transfer could mitigate tissue acidification and induce neuroprotection after focal cerebral ischemia in rats. In this study, the penumbral neurons were regulated by an optical-driven pump (archaerhodopsin/ArchT group) or channel (rhodopsin-2/ChR2 group). Neutral red fluorescence imaging was used to monitor the intracellular pH after ischemia, and the overall cerebral blood flow response was measured to evaluate neurological function. Their findings demonstrated that intracellular acidosis was mitigated by the optogenetic translocation of protons out of ArchT-expressing penumbral neurons 30 min after an ischemia event, showing therapeutic potential. Lately, another study further supports the potential neuroprotective effect of optogenetic technology following ischemic penumbra [81]. Using human induced pluripotent stem cells, researchers examined the electrophysiological activity of neuronal networks under controlled hypoxic conditions and found a decrease of their activity and synchronicity under low oxygen conditions. Photostimulation exhibited neuroprotective effects on neurons suffering hypoxia by maintaining or triggering the lactate shuttle through activating signaling molecules. In addition, photostimulation induced secretion of various factors and changes in neuronal cell activity, which also affected the outcomes of brain injury. For example, when the hippocampus of a rat carrying ChR2 was optically stimulated, the expression of Bcl-xL, an antiapoptotic protein, was upregulated in the neurons and surrounding cells [82].

Aside from neurons, autophagy and acidosis are also activated in various cell types after stroke, such as glial cells and microvascular cells. Optogenetics is known to impart cell type-specific control of glial cell activity with high spatiotemporal resolution. Therefore, Beppu et al. [83] attempted to reverse glial acidosis in hypoperfused tissue by activating ArchT and driving the outward proton pump using optical stimulation. In response, the release of glutamate was inhibited, and ischemic brain injury was alleviated in vivo, offering another approach that can potentially be used for neuroprotection.

In conclusion, these results show that optogenetic interventions targeting the acid–base balance and inhibiting nerve cell apoptosis can improve the survival ability of tissues balance, apoptosis or autophagy of neural cells are prospective for the treatment of brain injury.

### 3.4. Optogenetics in Promoting Regeneration

Brain injury may induce permanent neuron loss. Given the limited number of endogenous neural stem cells (NSCs), inducing regeneration in a damaged nervous system is challenging. However, neural transplantation (i.e., supplementing the injured brain with exogenous stem cells) may be a feasible approach. Transplanted stem cells achieve neuroprotection, immune regulation, and neuroregeneration through multiple mechanisms [84,85,86]. Optogenetic techniques combined with NSC-based therapies have the potential to enhance neuroregeneration and improve cell therapy outcomes [87].

Recent research has demonstrated that photostimulation of host cortico-spinal tract axons being regenerated into grafts elicited distinct and segregated neuronal network responses [86]. Optogenetic stimulation of graft-derived axons extending from the graft into the denervated spinal cord also triggered local host neuronal network responses, and behavioral stimulation elicited focal synaptic responses within grafts as shown by in vivo imaging [86]. In another study, optogenetic stimulation intact rat corticospinal tract post-stroke restores motor control by promoting axonal sprouting from the intact to the denervated cervical hemi-cord [88]. In addition, optogenetics has been used to drive the excitatory outputs of grafted NSCs and to increase forelimb use as well as motor activity on the stroke-affected side in a rat model [89].

In neural cell regeneration, recent work by Giraldo et al. [90] also indicated the marked potential of optogenetics in treating conditions such as SCI. In this study, blue light stimulation of NPCs engineered to ectopically express ChR2 (ChR2-NPCs) prompted an influx of cations and a subsequent increase in proliferation and differentiation from NPCs into oligodendrocytes and neurons. Further, stimulation drove the polarization of astrocytes from a pro-inflammatory to a pro-regenerative/anti-inflammatory phenotype. In another study, researchers used optogenetic techniques to control the activity of striatal neurons and investigated how their activity affected the survival and migration of transplanted NSCs as well as the overall neurological outcome after ischemic stroke [91]. They found that inhibitory stimulation of striatal neurons at 3–7 days post- ischemia led to increased neuroregeneration in the subventricular zone, bridging the gap between neuronal modulation and behavior of the NSCs [91]. While studies have been conducted to demonstrate the combined effect of stem cell-based therapy and optogenetics in stroke and SCI models, few have examined their effect in a TBI model. To the best of our knowledge, there has been only one study involving stem cell-based therapy with optogenetics in TBI [57]. In this study, the doublecortin, which is expressed by neuronal progenitor and postmitotic neuronal precursor cells, was coupled with ChR2-EGFP. Results showed that this ChR2-mediated depolarization approach promoted survival and maturation of newborn cells after TBI.

Combinatorial approaches using optogenetics with other advanced methods have also exhibited therapeutic promise. Hydrogel technology is currently used to study spinal cord repair and neuroregeneration [92,93]. Combined with light stimulation, injectable self-healing hydrogels provided shear forces that enhance the efficiency of a plasmid encoding bacteriorhodopsin, thus enabling the introduced NSCs to proliferate and differentiate smoothly [94].

## 4. Clinical Perspectives and Challenges

Currently, either conventional stimulation techniques or pharmacological therapies have some limitations in their clinical application. Optogenetic real-time monitoring and therapeutic interventions may be optimized for choice or provide a complementary treatment for the intervention of brain injury.

Optogenetics holds great promise for therapeutic application in clinics. Until now, an important achievement in optogenetic therapy was obtained in treating human retinitis pigmentosa- a neurodegenerative eye disease [95]. A serotype 2.7m8 adeno-associated virus (AAV) vector, encoding the light-sensing channelrhodopsin protein ChrimsonR fused with the red fluorescent protein tdTomato13, was administered by a single intravitreal injection into the patient’s worse-seeing eye to target retinal ganglion cells. Specially engineered goggles and a neuromorphic camera then converted images into light pulses that were projected onto the patient’s retina to activate modified retinal ganglion cells for visual tasks. Results showed that the previously blind patient could identify, count, locate and touch different objects with his post-treatment eyes when wearing the light-stimulating goggles [95]. In addition, prior research has reported that optogenetic stimulation could be applied in many other fields, including respiratory [96], muscle [97], and urinary systems [98]. All these results demonstrated the great value of the optogenetics in treating human disease or injury.

Despite broad clinical application prospects, there remain some important challenges associated with optogenetics-based therapies. 1. The main hindrance is the delivery of optogenetic tools in patient’s brain. This invasive operation may induce damage to brain tissue. In addition, weight/size of the implants and power cable connections, which may affect patients’ normal activities, also need to be considered. Recent advances in wireless technology and device miniaturization might partly solve this issue [99], while clinical application is still some way off. 2. Safety issue of the virus. Virus transduction is a widely used and effective method in optogenetic stimulation. The AAV is regarded as an ideal viral vector applied in gene therapy for many diseases [100,101,102]. However, a recent trial of AAV vector therapy has shown its possibility of inducing cancer [103]. The safety issue of gene therapy has once again been thrust into the public eye and needs to be carefully addressed. 3. Research on non-human primates (NHP) is still in its infancy. Compared to rodents, NHPs have larger brain volume and a more complicated central nervous system, all of which present great challenges for the translation of optogenetic study into a clinically effective therapy [104]. 4. As foreign antigens, opsins or viruses produce an underlying immune response, which may lead to neuronal death [105]. 5. Numerous ethical issues related to the application of optogenetics in humans, also need to be accounted for [106].

## 5. Conclusions

With high spatiotemporal precision, optogenetics has paved the way for its multiple applications in the neuroscience research and related translational medicine. In this review, we summarized the status quo of optogenetics and its application in the most recent brain injury research, including monitoring the brain, analyzing neural circuitry, protecting neural cells and promoting regeneration. Further, we reviewed the therapeutic potential and current challenges of this approach, aiming at advancing optogenetics in understanding and treating pathological conditions related to brain injury.

## Figures and Tables

**Table 1:** Commonly Used Optogenetic Tools.

Opsins	Description	Mode	Properties	Reference
ChR2	Cation channel responsive to blue light; commonly used for optogenetics	Excitatory	Millisecond temporal precision; a high risk of desensitization	Boyden et al., 2005 [21]
ChETA, ChIEF	Ultrafast opsin, site directed mutation and Chimeric modification of ChR2	Excitatory	Higher frequency activation and more rapid deactivation than ChR2	Lin et al., 2009 [22] Gunaydin et al., 2010 [23]
VChR1	Redshifted opsin with a similar photocurrent as ChR1	Excitatory	Slow photocurrent kinetics; low efficiency in high frequency stimulations	Zhang et al., 2008 [24]
C1V1	A chimeric combination of ChR1 and VChR2	Excitatory	High light sensitivity; good expression level on membranes	Hososhima et al., 2015 [25]
ReaChR	Mutant based on VChR1	Excitatory	Better opsin expression than VChR1; slow channel closing rate	Lin et al., 2013 [26]
NpHR	Chloride channel responsive to yellow light	Inhibitory	Millisecond temporal precision; poor trafficking to the membrane; unsuited for long-scale or high-quantity silencing	Nagel et al., 2003 [27]
eNpHR	Site directed mutation and chimeric modification of NpHR	Inhibitory	High-level expression with augmented inhibitory function; better opsin expression than NpHR; interfere with excitability of neurons	Gradinaru etal., 2008 [28] Ferenczi et al., 2012 [29]
Arch	Proton pump silences neurons in response to yellow light	Inhibitory	Good for large-scale silencing; high light sensitivity, photocurrents and expression levels	Chow et al., 2010 [30]

ChR2 denotes Channelrhodopsin-2, ChETA Channelrhodopsin-2 with E123T mutation, VChR1 Volvox carteri channelrhodopsin-1, ReaChR Red-activatable channelrhodopsin, NpHR Halorhodopsins, eNpHR enhanced NpHR, Arch Archaerhodopsin.

**Table 2:** Optogenetic TBI studies.

Research Topics	Model	Optogenetic Tools	Area	Reference
Microglia-mediated mechanisms underlying synaptic loss	Controlled cortical impact	Parvalbumin	CA1 hippocampus	Krukowski et al., 2021 [51]
Longitudinal changes in cortical motor map	Controlled cortical impact	ChR2	Motor cortex	Nguyen et al., 2021 [52]
Improvement of spatial recognition memory impairment	Controlled cortical impact	ArCh	RSC	Zeng et al., 2020 [53]
Response signals from cortical surfaces	Controlled cortical impact	ChR2	RSC	Zhang et al., 2018 [47]
Relationship between neuronal and vascular reactivity	Closed head injury	ChR2	Cortex, arterioles and venules in brain	Mester et al., 2021 [54]
Neuronal function following TBI	Closed head injury	ChR2	Peri-contusional brain tissue	Adams et al., 2018 [55]
The structural reorganization of axonal projection terminals and the functional activity of the thalamocortical network	Fluid percussion injury	ChR2	S1	Ndode-Ekane et al., 2021 [56]
Survival and maturation of newborn neurons during adult neurogenesis	Fluid percussion injury	ChR2	DG hippocampus	Zhao et al., 2018 [57]

CA1 hippocampus denotes Cornu Ammonis subfield 1 in hippocampus, RSC Retrosplenial Cortex, S1 Primary somatosensory cortex, DG hippocampus Dentate gyrus in hippocampus.

## Data Availability

Not applicable.

## Abbreviations

1P	One-photon
2P	Two-photon
AAV	Adeno-associated virus
ABI	Acute brain injury
Arch	Archaerhodopsin
BiPOLES	Bidirectional pair of opsins for light-induced excitation and silencing
ChETA	Channelrhodopsin-2 with E123T mutation
ChR2	Channelrhodopsin-2
ChRs	Channelrhodopsins
eNpHR	Enhanced NpHR
GAP43	Growth-associated factor 43
iM1	Ipsilesional primary motor cortex
LCN	Lateral cerebellar nucleus
LED	Light-emitting diode
mTBI	Mild traumatic brain injury
NHP	Non-human primates
NpHR	Halorhodopsins
ReaChR	Red-activatable channelrhodopsin
RSC	Retrosplenial cortex
SCI	Spinal cord injury
TBI	Traumatic brain injury
VChR1	Volvox carteri channelrhodopsin-1
